# Decreased plasma concentrations of kynurenine and kynurenic acid in schizophrenia patients

**DOI:** 10.1192/j.eurpsy.2023.658

**Published:** 2023-07-19

**Authors:** M. Markovic, M. Stasevic, S. Ristic, M. Stojkovic, M. Velimirović Bogosavljević, T. Stojkovic, M. Zivkovic, I. Stasevic Karlicic, S. Totic Poznanovic, T. Nikolic, N. Petronijevic

**Affiliations:** ^1^ Clinic for mental disorders “Dr Laza Lazarevic”; ^2^Institute for medical and clinical biochemistry, Belgrade, Serbia; ^3^Clinic for Neurology, Clinical Center of Nis, Nis; ^4^Faculty of Medicine, University of Belgrade; ^5^Institute for medical and clinical biochemistry, Belgrade; ^6^Faculty of Medicine, University of Priština – Kosovska Mitrovica, Kosovska Mitrovica; ^7^Clinic for psychiatry, University Clinical Centre of Serbia, Belgrade, Serbia

## Abstract

**Introduction:**

The kynurenine pathway of tryptophan catabolism has come into the spotlight of schizophrenia research since its catabolites exert neuroactive effects. A strong body of evidence suggests that kynurenic acid, a catabolite of kynurenine pathway, acts as the only endogenous NMDA receptor antagonist leading to the weakening of circuits in layer III of dorsolateral prefrontal cortex of schizophrenia patients. Studies exploring the levels of kynurenic acid and other metabolites of tryptophan in peripheral blood did not yield any definite conclusions.

**Objectives:**

Primary objective of this study was to assess differences in concentrations of key constituents of kynurenic pathway in blood plasma – tryptophan (TRP), kynurenine (KYN) and kynurenic acid (KYNA) between schizophrenia patients (SCZ) and healthy controls (HC). Secondary objective was to explore correlations between these concentrations and clinical characteristics.

**Methods:**

In our two-centre prospective case-control study we measured plasma concentrations of TRP, KYN and KYNA in 36 healthy controls (HC) and 38 schizophrenia (SCZ) patients during acute exacerbation and remission and explored the correlations with clinical parameters using PANSS scale. The patients were matched with HC by age, sex and body mass index and exclusion criteria included obesity class 2 or higher, any concomitant organic mental or neurological disorder, acute or chronic inflammatory disease, and use of immunomodulatory drugs or psychoactive substances.

**Results:**

TRP concentrations were significantly higher in HC than in SCZ patients in acute phase (p<0,001) and remission (p<0,001), while SCZ patients in acute phase had significantly higher TRP levels than in remission (p<0,01). Levels of KYNA and KYN were significantly lower in SCZ patients than in HC both in acute phase and remission, all with high statistical significance (p<0,001). There was no statistically significant difference between acute phase and remission neither for KYN (p>0,05), nor for KYNA (p>0,05). There was no correlation of plasma levels of TRP, KYN and KYNA with total PANSS score, PANSS positive scale score, PANSS negative scale score and PANSS general psychopathology scores, both in acute phase and remission (p>0,05). Also, there was no correlation between plasma levels of TRP, KYN and KYNA in SCZ patients in remission with improvements measured with PANSS scale (p>0,05).

**Conclusions:**

Although there are concerns about the value of measurement of metabolites of kynurenine pathway in the peripheral blood, our data suggest that significantly decreased levels of KYN and KYNA could suggest that disrupted TRP degradation in SCZ patients may be reflected in the peripheral blood as well. Further studies of peripheral levels of kynurenine pathway metabolites on larger samples should also explore effects of antipsychotic therapy, but also their correlation with other clinical parameters such as neurocognition.

**Disclosure of Interest:**

None Declared

